# Dutogliptin in Combination with Filgrastim in Early Recovery Post-Myocardial Infarction—The REC-DUT-002 Trial

**DOI:** 10.3390/jcm11195728

**Published:** 2022-09-27

**Authors:** Dirk von Lewinski, Martin Benedikt, Hannes Alber, Jan Debrauwere, Pieter C. Smits, István Édes, Róbert Gábor Kiss, Béla Merkely, Gergely Gyorgy Nagy, Pawel Ptaszynski, Maciej Zarebinski, Jacek Kubica, Andrzej Kleinrok, Andrew J. S. Coats, Markus Wallner

**Affiliations:** 1 Clinical Department of Cardiology, Medical University of Graz, 8036 Graz, Austria; 2 Clinic of Klagenfurt at Wörthersee, 9020 Klagenfurt, Austria; 3 Algemeen Stedelijk Ziekenhuis, 9300 Aalst, Belgium; 4 Department of Cardiology, Maasstad Ziekenhuis Rotterdam, 3079 Rotterdam, The Netherlands; 5 Debreceni Egyetem Klinikai Központ Regionális és Intézményi Kutatásetikai Bizottság, Pf. 12, 4012 Debrecen, Hungary; 6 Magyar Honvédség Egészségügyi Központ Intézményi és Regionális Kutatásetikai Bizottság, Róbert Károly körút 44, 1134 Budapest, Hungary; 7 Heart and Vascular Center, Semmelweis University, 1122 Budapest, Hungary; 8 Borsod-Abauj-Zemplen County Central Hospital and University Teaching Hospital, 1st Department of Internal Medicine and Cardiology, 3526 Miskolc, Hungary; 9 Department of Electrocardiology, Central University Hospital, Medical University of Lodz, 92-213 Lodz, Poland; 10 SPS Szpital Zachodni im. sw. Jana Pawła II, Invasive Cardiology Department, Daleka 11, 05-825 Grodzisk mzowiecki, Poland; 11 Department of Cardiology, Collegium Medicum, Nicolaus Copernicus University, 85-094 Bydgoszcz, Poland; 12 Academy of Zamosc, Institute of Humanities and Medicine, Pereca 2 St., 22-400 Zamosc, Poland; 13 Heart Research Institute, 7 Eliza St, Newtown, NSW 2042, Australia; 14 Cardiovascular Research Center, Lewis Katz School of Medicine, Temple University, Philadelphia, PA 19140, USA

**Keywords:** myocardial infarction, regeneration, safety, tolerability, G-CSF, DPP-4, cMRI

## Abstract

Patients with acute myocardial infarction are at high risk for developing heart failure due to scar development. Although regenerative approaches are evolving, consistent clinical benefits have not yet been reported. Treatment with dutogliptin, a second-generation DPP-4 inhibitor, in co-administration with filgrastim (G-CSF) has been shown to enhance endogenous repair mechanisms in experimental models. The REC-DUT-002 trial was a phase 2, multicenter, double-blind placebo-controlled trial which explored the safety, tolerability, and efficacy of dutogliptin and filgrastim in patients with ST-elevation Myocardial Infarction (STEMI). Patients (*n* = 47, 56.1 ± 10.7 years, 29% female) with STEMI, reduced left ventricular ejection fraction (EF ≤ 45%) and successful revascularization following primary PCI were randomized to receive either study treatment or matching placebo. Cardiac magnetic resonance imaging (cMRI) was performed within 72 h post-PCI and repeated after 3 months. The study was closed out early due to the SARS-CoV-2 pandemic. There was no statistically significant difference between the groups with respect to serious adverse events (SAE). Predefined mean changes within cMRI-derived functional and structural parameters from baseline to 90 days did not differ between placebo and treatment (left ventricular end-diastolic volume: +13.7 mL vs. +15.7 mL; LV-EF: +5.7% vs. +5.9%). Improvement in cardiac tissue health over time was noted in both groups: full-width at half-maximum late gadolinium enhancement (FWHM LGE) mass (placebo: −12.7 g, treatment: −19.9 g; *p* = 0.23). Concomitant treatment was well tolerated, and no safety issues were detected. Based on the results, the FDA and EMA have already approved an adequately powered large outcome trial.

## 1. Introduction

ST-elevation myocardial infarction (STEMI) is an acute life-threatening disease and a major health burden. While the advent of primary percutaneous intervention (PCI) has drastically improved survival rates in patients with STEMI, a significant percentage of patients still develop heart failure (HF), leading to adverse long-term clinical outcomes [1,2]. Although pathophysiological mechanisms underlying STEMI have been identified in animal models, treatment remains challenging in the clinical setting due to various interdependent mechanisms involved in the progression of LV-dysfunction. The first wave of damage is caused by ischemia and once blood flow is restored, a second wave of injury occurs during reperfusion. 

**Figure 1:**
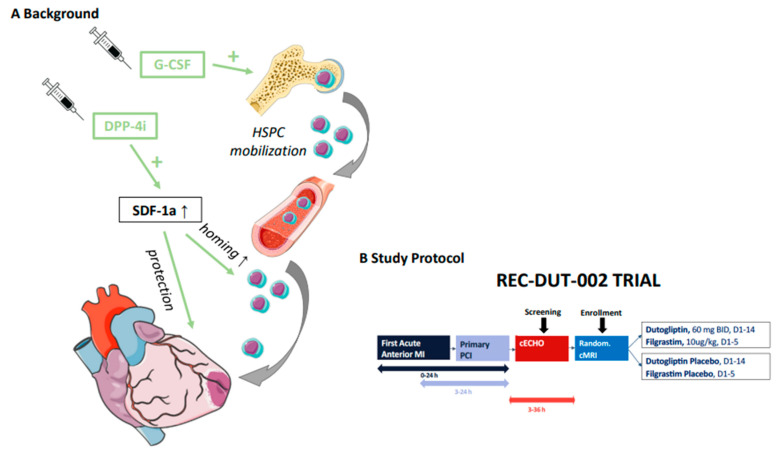
(**A**) The strategy consists of sustained mobilization of endogenous hematopoietic stem and progenitor cells (HSPC) into circulating blood with granulocyte colony-stimulating factor (G-CSF, filgrastim) and inhibition of dipeptidyl peptidase 4 (DPP-4; dutogliptin), which degrade the stem cell binding chemokine to sustain stem cell recruitment and mobilization to the injured myocardium. (**B**) Subjects received study treatment within 36 hours after stent implantation. Study treatment consisted of dutogliptin 60 mg or matching placebo was administered twice daily by subcutaneous injection for 14 days. Filgrastim 10 μg/kg or matching placebo was co-administered for the first 5 days. The total duration of study participation for each subject was 3 months.

Promising results from early stem cell trials have not been confirmed in larger multicenter randomized controlled studies due to poor cell survival, retention, and sustained activity in the infarcted heart—a critical requirement for effective treatment [3,4,5]. To enable stem cells to exert a therapeutic benefit in a clinical setting more effectively, an alternative strategy was developed which does not require harvest and reinfusion but instead relies on the intrinsic homing and migration of mobilized endogenous stem cells to the injured area. This strategy consists of sustained mobilization of endogenous stem cells into circulating blood with granulocyte colony-stimulating factor (G-CSF) in combination with inhibition of dipeptidyl peptidase 4 (DPP-4) (Figure 1).

In STEMI, the potential of G-CSF to improve myocardial function and survival in patients was first investigated in the G-CSF-STEMI trial, revealing a positive influence on myocardial perfusion if G-CSF was given early, but no overall improvement of myocardial function and survival was reported when used as a monotherapy [6]. However, no major safety issues were identified with this approach. This observation was confirmed in a meta-analysis of eight eligible studies using G-CSF (*n* = 385 patients) in patients with myocardial infarction [7]. However, a more recent CMR sub-study of the STEM-AMI OUTCOME trial revealed improved LV function 6 months post-STEMI when treated with G-CSF [8]. This trial successfully initiated treatment early within 24 h after PCI.

Besides its anti-diabetic effects, a large number of bioactive molecules can be cleaved by DPP-4. After STEMI, DPP-4 inhibition will prevent the degradation of stromal cell-derived factor 1 alpha (SDF-1a), which has been identified as key regulator in stem cell homing to ischemic and injured myocardium [9]. SDF-1a is also thought to confer direct protection against ischemia-reperfusion (IR) injury. It exerts pleiotropic effects on ischemic myocardium such as gradient-guided homing of stem cells towards sites of myocardial injury and direct protection via intracellular pro-survival signal transduction pathways [10].

As both therapeutic strategies (G-CSF, DPP-4 inhibition) do not provide a significant benefit when used as monotherapy, the concept of administering them simultaneously was investigated. Preclinical studies demonstrated that the treatment with various DPP-4 inhibitors including dutogliptin along with G-CSF significantly reduced mortality and improved hemodynamic parameters [11,12]. 

Due to these results, we initiated the multicenter, randomized, double-blind, placebo-controlled REC-DUT-002 trial to explore the safety, tolerability, and efficacy of dutogliptin and co-administration with filgrastim (G-CSF) in patients with STEMI following successful PCI and stent implantation.

## 2. Methods

The ethics review board at each participating centre approved the protocol, and the trial was performed in accordance with the Declaration of Helsinki. The study was registered at clinicaltrials.gov (NCT NCT03486080).

Detailed inclusion and exclusion criteria as well as the study design of the Rec-DUT-002 trial has been published elsewhere [13]. In short, patients 18 to 85 years of age with successfully treated STEMI (PCI within 24 h after symptom onset) but reduced LV-EF (≤45%) were eligible. Eligibility was evaluated in a stepwise manner: Medical history, physical examination, and safety laboratory screening tests were completed prior to conducting the cardiac echocardiogram (cECHO). After successful screening and providing written informed consent, patients were randomized (stratified by study site) using an interactive web response system (IRT). Subjects received study treatment within 36 h of stent implantation. Study treatment consisted of dutogliptin (60 mg) or matching placebo and was administered twice daily by subcutaneous (SC) injection for 14 days. Additionally, filgrastim 10 μg/kg or matching placebo was co-administered with the dutogliptin daily for the first 5 days via SC injections. The total duration of study participation for each subject was 3 months.

Safety assessments were performed on Day 0, Day 1 (baseline), Day 2, Day 3, Day 5, Day 15, and Day 90. Safety assessments included physical examinations, vital signs, laboratory tests, ECG, and documentation of adverse events (AEs). Abnormal laboratory results for liver enzymes triggered expedited reporting according to the following thresholds: ALT or AST > 8xULN, or ALT or AST > 3xULN and (TBL > 2xULN, or INR > 1.5), or ALT or AST > 3xULN with the appearance of fatigue, nausea, vomiting, right upper quadrant pain or tenderness, fever, rash, and/or eosinophilia (>5%).

Efficacy assessments were performed within 72 h after PCI (baseline) and on Day 90 to analyze cardiac function (cardiac magnetic resonance imaging (cMRI)). cMRI scans were performed according to the standard protocol provided to sites by the MRI core laboratory. Both 1.5 and 3 Tesla scanners were used during the study. The serial cMRIs obtained for each subject were performed using the same scanner. The cMRI scan was performed within 72 h after PCI. If a randomized subject prematurely discontinued study participation prior to the Day 90 visit, a second cMRI scan was performed during the Early Termination visit. At baseline and Day 90, left and right ventricular structural and function indices such as ejection fraction (EF), end-systolic volume (ESV), end-diastolic volume (EDV), infarct size (late gadolinium enhancement (LGE), full-width at half-maximum late gadolinium enhancement mass (FWHM LGE)) and left ventricular mass were assessed via blind review of cMRI scans.

## 3. Objectives

The primary study objective was to evaluate the safety and tolerability of dutogliptin when co-administered with filgrastim in patients with STEMI compared with placebo. Safety assessments included reported treatment-emergent AEs (TEAE), clinical laboratory tests, electrocardiograms (ECGs), vital signs, and physical examinations. The secondary objectives of the study were to explore the efficacy of this treatment including changes in cardiac function and structure (cMRI) from baseline to Day 90: predefined parameters were LVEF, left ventricular–end-systolic volume (LVESV; absolute and indexed), left ventricular—end-diastolic volume (LVEDV; absolute and indexed), infarct size (FWHM LGE mass) and left ventricular mass (absolute and indexed).

Moreover, predefined individual clinical safety endpoints included recurrent non-fatal myocardial infarction, non-fatal stroke, death due to any cause, cardiovascular death (death due to acute myocardial infarction, chronic heart failure [CHF], stroke, or sudden cardiac death), stent thrombosis or CHF hospitalization as well as a composite clinical endpoint (MACE), including non-fatal myocardial infarction, non-fatal stroke, cardiovascular death, stent thrombosis, and CHF hospitalization. Measurement of biomarkers (N-terminal pro-b-type natriuretic peptide [NT-proBNP] and high sensitivity troponin) were optional.

## 4. Statistical Analysis

The aim of the study was to prove safety of the combined treatment of G-CSF and dutogliptin. The sample size was selected primarily due to feasibility arguments, but power calculations were performed to demonstrate the effect size in terms of safety and efficacy, which could be expected to be observed in this study. Our calculation indicated that with 70 subjects per treatment group, one would only detect significant differences if AE rates were 15.2% for dutogliptin in co-administration with filgrastim versus 1% for the placebo. With regards to the potential therapeutic effects of the comprehensive cMRI assessment, 140 subjects (110 subjects excluding dropouts) would be sufficient to detect differences of 3.8% in mean change in LVEF from baseline to 90 days, with an expected standard deviation of 7.0, two-sided 5% confidence interval and 80% power. The sample size used is considered appropriate for our study, even though it has not been formally calculated for the primary endpoint.

The intention-to-treat (ITT)-population included all randomized subjects (N = 48). The per-protocol (PP) population included all randomized subjects who had completed treatment with IMP, had a cMRI at baseline and Day 90 without protocol deviations relevant for efficacy analysis. Decisions on all protocol violations were made on a case-by-case decision in a blinded data review meeting before database closure. The efficacy evaluations are based on the PP population. The safety population included all randomized subjects who had received at least one dose of IMP (N = 47). 

Changes from baseline to Day 90 in cardiac function parameters, infarct size, left ventricular mass and regional wall motion were evaluated using an analysis of covariance model with randomization stratification factors as covariates. The Wilcoxon test for unpaired observations was used to compare groups. Frequencies of individual and combined clinical endpoints on Day 15 and Day 90 are summarized in frequency tables. Logistic regression models were applied using the randomization stratification factors as covariates. In addition, differences between the two treatment groups were tested for statistical significance using Fisher’s exact test. Time to cardiovascular event was descriptively analyzed using the Kaplan–Meier method. Median time to cardiovascular event per treatment group and the hazard ratio between the two treatment groups was calculated along with 95% confidence intervals. A Cox regression model was applied for the time to cardiovascular event with randomization stratification factors as covariates. A log-rank test was conducted to test the significance between treatment groups.

At the start of the COVID-19 pandemic in 2020, the EMA recommended halting clinical studies, so enrollment for this study was paused for 4 months at all study sites. When reopened, enrollment initially accelerated, but was then extremely slow during the next wave in late 2020. Fewer qualifying patients were admitted to hospitals, and of those qualifying, an even smaller subset was willing to give informed consent due to concerns of staying in the hospital longer than necessary, and not wanting to have home visits by qualified nurses during the ongoing pandemic as before. In addition, sites were unable to dedicate staff for data collection and patient follow-up as required in the protocol.

## 5. Results

**Figure 2:**
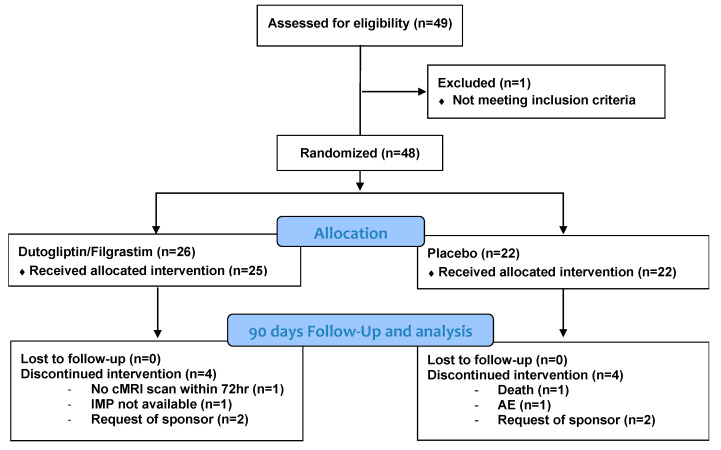
Diagram of patients included in the current study. AE = adverse event, cMRI = cardiac magnetic resonance imaging, IMP = investigational medicinal product.

A total of 140 patients were initially planned for enrollment. However, the study was closed out early due to the SARS-CoV-2 pandemic. In total, 49 patients were enrolled, with 48 subjects randomized, and 47 subjects received the study injections (25 in the treatment group and 22 in the placebo group) (Figure 2). Upon discharge from the hospital, adequate supplies of investigational medicinal products (IMPs) to complete all dosing were issued to the homecare nursing service, which administered all remaining doses to the subject at home. All subjects received dutogliptin, filgrastim, or placebo as planned from Day 1 to Day 14 except for the second dutogliptin/placebo dose on Day 3 (received by 44 of 47 subjects), Day 4 (received by 43 of 44 subjects), Day 11 (received by 41 of 42 subjects). Four patients in each group were terminated early from the study. Within the active group this was due to: request of the sponsor (2 patients), cMRI scan not available within 72 h after PCI (1 patient) and not enough IMP was available at the site of randomization (1 patient). Within the placebo group, the four early terminations were due to: request of the sponsor (2 patients), study termination (1 patient), and death (1 patient). Baseline characteristics are outlined in Table 1 and concomitant medication is displayed in Table 2.

None of the patients experienced non-fatal stroke, stent thrombosis or cardiovascular death, and there was no statistically significant difference between the active and placebo groups for non-fatal myocardial infarction (active group 2, placebo 1; *p* = 0.50), hospitalization due to chronic heart failure (*p* = 1.00), death due to any (other) cause (*p* = 0.47) or the combined clinical endpoints (*p* = 0.61). Reported SAEs were also not significantly different between the groups. No SAEs were found to be related to dutogliptin or filgrastim. The only SAEs experienced by more than one patient were pneumonia (4 [8.5%] patients/2 in each group) and acute myocardial infarction (3 [6.4%] 2 patients in treatment group and 1 patient in placebo group). One myocardial infarction was classified as myocardial infarction with nonobstructive coronary arteries (MINOCA), the second as recurrent myocardial infarction and the third occurred on day 1 as a peri-interventional myocardial infarction. SAEs are shown in Table 3. No early termination was related to the study drug treatment and no subject died following administration of dutogliptin + filgrastim treatment. 

One subject in the placebo group had a clinically relevant heart rate finding on Day 2, and another subject had an elevated temperature on Day 3 and Day 5. One subject in the active group had fever, which was considered clinically relevant at Day 1. One subject in the active group had a clinically relevant physical examination of injection site/draining node finding at Day 15. There were no clinically relevant differences between treatment groups regarding physical examinations, electrocardiograms (ECGs), or vital signs.

All laboratory safety tests were within acceptable limits, and there were no statistical differences between treatment groups. There were no evident patterns in absolute values or changes from baseline on any of the assessment days regarding clinical chemistry, hematology, and quantitative urinalysis in either treatment group. Elevated liver enzyme values at the start of the treatment rapidly returned to normal following the PCI and they were determined to be unrelated to dutogliptin or filgrastim.

Next, we looked at prespecified functional and structural cardiac changes from baseline to 90 days using cMRI scans (Table 4). Changes in left and right ventricular parameters over time did not differ significantly between the groups. Increases in mean change from Day 3 (baseline) to Day 90 values were seen in both groups for LVEDV (placebo: 13.7 ± 27.5 mL, treatment: 15.7 ± 28.1 mL), LVEDVI (placebo: 8.4 ± 15.3 mL/m^2^, treatment: 7.7 ± 14.1 mL/m^2^), and LVEF (placebo: 5.7 ± 7.8%, treatment: 5.9 ± 8.9%). Decreases in mean change from baseline were seen for LV mass (placebo: −16.1 ± 24.5 g, treatment: −15.1 ± 11.4 g) and LV mass index (placebo: −8.3 ± 14.2%, treatment: −7.9 ± 5.9%). Likewise, right ventricular parameters were also not significantly different between the two groups. However, RVEF did only increase in the treatment group (placebo: −0.3 ± 11.8%, treatment: 2.7 ± 6.4%). 

**Figure 3:**
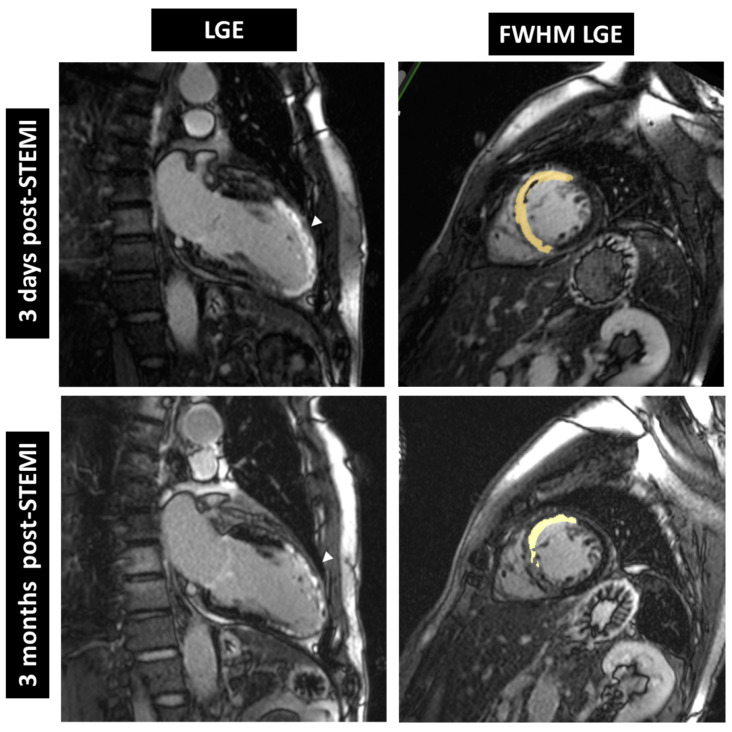
Cardiac magnetic resonance images from a patient receiving dutogliptin + filgrastim. LGE (left) and FWHM LGE (right) were reduced 3 months post-STEMI compared to baseline (3 days post-STEMI). LGE = late gadolinium enhancement, FWHM = full-width at half-maximum, arrow = LGE.

Improvement in cardiac tissue health over time was analyzed by cMRI LGE and full-width at half-maximum late gadolinium enhancement (FWHM LGE) as shown in Figure 3. FWHM LGE mass is a robust, semi-automated method to assess infarct size. The reduction in absolute FWHM LGE mass (INF; placebo: −12.7 ± 17.2 g, treatment: −19.9 ± 16.9 g; *p* = 0.23) and relative FWHM LGE mass (INF/VV; placebo: −6.6 ± 10.1, treatment: −12.7 ± 14.6%; *p* = 0.24) was more pronounced in the treatment group compared to placebo. However, this difference did not reach statistical significance (also see Table 5).

As biomarker analysis was optional and no central lab was established for this study, the collected data was not sufficient to pursue dedicated analysis.

## 6. Discussion

Previous clinical trials using stem cells have either shown modest results [4,14,15] or only moderate improvement of left ventricular function in smaller trials [16]. Another trial addressed SDF1a-mediated repair and healing processes using G-CSF and DPP-4 inhibitors [17] with neutral effects, whereas long-term follow-up data recently presented for the RIGENERA trial appeared promising 10 years after G-CSF therapy [18]. However, the patient number was very limited, and the pathophysiological rationale of these approaches remains intriguing. 

Myocardial infarction is a life-threatening event, and all treatments must have thoroughly evaluated safety profiles. Therefore, the present trial focused on the evaluation of clinical safety of the combined therapy with filgrastim and dutogliptin.

The frequency of TEAEs (Table S1), including subgroup analysis of serious, severe or related TEAEs, were similar in both groups. No subjects experienced related serious or severe TEAEs. Based on these findings, the treatment protocol can be considered safe in patients suffering large STEMI with reduced LV function. In addition, no TEAEs led to withdrawal of any treatment and no TEAEs were considered related to dutogliptin or filgrastim. Moreover, no TEAEs judged to be related to IMP were of severe intensity. 

The only SAE experienced by more than one patient was pneumonia, with two patients in each group not judged to be related to the study medication. Lastly, laboratory safety tests were all within acceptable limits, and there were no statistical differences between treatment groups. There were no evident patterns in absolute values or changes from baseline versus Day 90 on any of the assessment days regarding clinical chemistry, hematology, and quantitative urinalysis in either treatment group. 

While initiating a therapy before the onset of an MI would maximize the beneficial effects of the treatment, the occurrence of an MI is inherently unpredictable. Therefore, being able to initiate treatment as soon as possible post-MI is imperative. Previous trials reported average times from index myocardial infarction to randomization of 3 to 7 days [19]. However, for this trial, treatment initiation had to be started within 36 h after PCI. Although not powered for an efficacy endpoint, this delay may still be too long, which might have hampered positive efficacy outcomes. Therefore, unaltered LV function parameters are only descriptive. Overall LV dimensions increased moderately but were paralleled by decreasing LV mass in both groups. Likewise, LVEF recovered considerably in both groups from baseline to day 90. Similarly, no significant changes could be detected with respect to the right ventricle.

FWHM LGE mass was analyzed as a central tissue characterization parameter. Although no statistically significant differences were identified, as depicted in Table 5, for absolute FWHM LGE Mass, relative FWHM LGE Mass and border zone mass (2SD-5SD), all parameters tended to develop more pronounced effects in the treatment group. FWHM is considered the most reproducible method for infarct sizing in acute myocardial infarction [20].

## 7. Conclusions

Due to the COVID-19 pandemic, study recruitment was concluded earlier than planned, resulting in a smaller sample size. However, the trial was designed as an exploratory and not a confirmatory trial. Concomitant treatment of dutogliptin with filgrastim was well tolerated, and no safety issues were detected. Treatment-emergent AEs were seen with similar frequencies in the active and placebo group (overall, related, or serious AEs) and there were no deaths or withdrawals in the treatment group compared to one death in the placebo group.

Considering the morbidity and quality of life lost following the occurrence of a STEMI [21] and the excellent safety profile of dutogliptin along with data suggesting potential positive effects on cardiac function, studying this therapeutic approach in a large, adequately powered, NDA-enabling study is warranted. A large outcome trial evaluating treatment of dutogliptin in co-administration with filgrastim is already pre-approved by the FDA and EMA.

## Figures and Tables

**Table 1:** Summary of Baseline Characteristics (ITT Population).

Characteristic.	Active Group (N = 26)	Placebo Group (N = 22)	All (N = 48)
**Age at Screening [years; mean (SD)]**	55.1 (9.4)	57.2 (12.1)	56.1 (10.7)
**Female *n* (%)**	8 (30.8)	6 (27.3)	14 (29.2)
**Race**			
White *n* (%)	25 (96.2)	22 (100)	47 (97.9)
Other *n* (%)	1 (3.8)	0 (0.0)	1 (2.1)
**BMI [mean (SD)]**	27.3 (4.2)	26.1 (4.1)	26.7 (4.1)
**EF at Baseline [%; mean (SD)]**	39 (5)	39 (6)	39 (5)
**Medical History**			
Hyperlipidaemia *n* (%)	3 (11.5)	2 (9.1)	5 (10.4)
Type 2 diabetes mellitus *n* (%)	3 (11.5)	3 (13.6)	6 (12.5)
Obesity *n* (%)	2 (7.7)	3 (13.6)	5 (10.4)
Hyperuricemia *n* (%)	1 (3.8)	1 (4.5)	2 (4.2)
Arterial Hypertension *n* (%)	8 (30.8)	8 (36.4)	16 (33.3)
Previous acute myocardial infarction *n* (%)	1 (3.8)	1 (4.5)	2 (4.2)
Tobacco abuse *n* (%)	3 (11.5)	2 (9.1)	5 (10.4)
Depression *n* (%)	3 (11.5)	1 (4.5)	4 (8.3)
Chronic obstructive pulmonary disease *n* (%)	3 (11.5)	0 (0.0)	3 (6.3)

ITT = intention-to-treat, N = number of subjects with events, percentages based on N, SD = standard deviation, BMI = body mass index.

**Table 2:** Summary of Concomitant Medications (ITT Population).

Concomitant Medication	Active Group (N = 26) *n* (%)	Placebo Group (N = 22) *n* (%)	ITT Population (N = 48) *n* (%)
**ACEi inhibitors/ARBs**	26 (100)	22 (100)	48 (100)
**Antithrombotic agents**	26 (100)	22 (100)	48 (100)
**Beta-Blockers**	24 (92.3)	22 (100)	46 (95.8)
**Lipid modifying agents**	24 (92.3)	22 (100)	46 (95.8)
**Drugs for acid related disorders**	23 (88.5)	20 (90.9)	43 (89.6)
**Potassium-sparing agents**	12 (46.2)	15 (68.2)	27 (56.3)
**Loop diuretics**	7 (26.9)	8 (36.4)	15 (31.3)

ITT = intention-to-treat, N = number of subjects with events, percentages based on N, ACE = angiotensin-converting enzyme, ARB = angiotensin receptor blockers.

**Table 3:** Severe and Serious Treatment-Emergent Adverse Events (Safety Population).

	Active Group (N = 25) *n* (%)	Placebo Group (N = 22) *n* (%)	Safety Population (N = 47) *n* (%)
**Any serious TEAE**	5 (20.0)	4 (18.2)	9 (19.1)
**Cardiac disorders**	4 (16.0)	3 (13.6)	7 (14.9)
Acute myocardial infarction	2 (8.0)	1 (4.5)	3 (6.4)
Angina pectoris	0 (0.0)	1 (4.5)	1 (2.1)
Acute cardiac failure	1 (4.0)	0 (0.0)	1 (2.1)
Coronary artery stenosis	1 (4.0)	0 (0.0)	1 (2.1)
**Pneumonia**	2 (8.0)	2 (9.1)	4 (8.5)
**Acute Kidney Injury**	0 (0.0)	1 (4.5)	1 (2.1)

N = number of subjects with events, percentages based on N, TEAE = treatment-emergent adverse event.

**Table 4:** Summary of Mean Cardiac Magnetic Resonance Imaging Results for Left and Right Ventricular Parameters: Change from Day 3 to Day 90 (ITT and PP Populations).

Parameter	ITT Population Mean (SD) *n*		PP Population Mean (SD) *n*	
	Active Group	Placebo Group	Active Group	Placebo Group
LV-EDV [ml]	15.7 (28.09) 20	13.7 (27.50) 14	17.4 (27.79) 19	13.7 (27.50) 14
LV-EDVI [ml/m^2^]	7.7 (14.09) 20	8.4 (15.31) 14	8.6 (13.89) 19	8.4 (15.31) 14
LV-ESV [ml]	1.0 (25.95) 20	−1.2 (26.33) 14	2.6 (25.59) 14	−1.2 (26.33) 14
LV-ESVI [ml/m^2^]	0.4 (12.87) 20	0.1 (14.74) 14	1.3 (12.57) 19	0.1 (14.74) 14
LV-Mass [g]	−15.1 (11.42) 20	−16.1 (24.48) 14	−14.4 (11.26) 19	−16.1 (24.48) 14
LV-Mass Index [%]	−7.9 (5.89) 20	−8.3 (14.23) 14	−7.4 (5.73) 19	−8.3 (14.23) 14
LV-EF [%]	5.9 (8.86) 20	5.7 (7.81) 14	5.2 (8.52) 19	5.7 (7.81) 14
RV-EDV [ml]	22.7 (32.45) 20	16.2 (23.19) 14	22.1 (33.23) 19	16.2 (23.19) 14
RV-EDVI [ml/m^2^]	11.4 (16.70) 20	9.3 (11.57) 14	11.0 (17.06) 19	9.3 (11.57) 14
RV-ESV [ml]	6.7 (13.06) 20	7.0 (23.52) 14	6.5 (13.39) 19	7.0 (23.52) 14
RV-ESVI [ml/m^2^]	3.4 (6.73) 20	0.1 (7.20) 14	3.2 (6.89) 19	0.1 (7.20) 14
RV-EF [%]	2.7 (6.37) 20	−0.3 (11.80) 14	2.8 (6.50) 19	−0.3 (11.80) 14

ITT = intent to treat, PP = per protocol, SD = standard deviation, *n* = number of subjects with available result, EDV = end diastolic volume, EDVI = end diastolic volume index, ESV = end systolic volume, ESVI = end systolic volume index, EF = ejection fraction.

**Table 5:** Summary of Tissue Characterization Parameters from Day 3 to Day 90 (ITT and PP Populations).

Parameter	ITT Population Mean (SD) *n*		PP Population Mean (SD) *n*	
	Active Group	Placebo Group	Active Group	Placebo Group
FWHM LGE Mass (INF) [g]	−19.9 (16.93) 21	−12.7 (17.23) 14	−20.1 (17.04) 19	−12.7 (17.23) 14
Relative FWHM LGE Mass (INF/VV) [%]	−12.7 (14.58) 20	−6.6 (10.06) 14	−13.3 (10.06) 19	−6.6 (14.74) 14
Border zone Mass (2SD-5SD) [g]	−0.3 (2.57) 21	0.2 (6.34) 14	−0.5 (2.55) 19	0.2 (6.34) 14

ITT = intention-to-treat, PP = per protocol, SD = standard deviation, *n* = number of subjects with available result, FWHM LGE = full-width at half-maximum late gadolinium enhancement, INF = absolute myocardial infarction size, VV = ventricular volume, 2SD = 2 Standard deviation, *p*-values obtained via Wilcoxon test.

## Data Availability

The data presented in this study are available on request from the corresponding author.

## Supplementary Materials

The following supporting information can be downloaded at: https://www.mdpi.com/article/10.3390/jcm11195728/s1, Table S1: Treatment-Emergent Adverse Events (Safety Population).
