# Supplementary material for: Dutogliptin in Combination with Filgrastim in Early Recovery Post-Myocardial Infarction—The REC-DUT-002 Trial
Source: J Clin Med. 2022 Sep 27;11(19):5728. doi: 10.3390/jcm11195728 (PMC9571810; doi:10.3390/jcm11195728)
Supplement: Supplementary file 1 [file jcm-11-05728-s001.zip › jcm-1850995-supplementary.pdf]

Supporting Information

The intention-to-treat (ITT)-population included all randomized subjects (N=48). The Per Protocol (PP) Population included all randomized subjects who had completed treatment with IMP, had a cMRI at baseline and Day 90 without protocol deviations relevant for efficacy analysis. Decisions on all protocol violations were made on a case-by-case decision in a blinded data review meeting before database closure. The efficacy evaluations are based on the PP-population. The Safety Population included all randomized subjects who had received at least one dose of IMP (N=47).

Changes from baseline to Day 90 in cardiac function parameters, infarct size, LV mass and regional wall motion were evaluated using an analysis of covariance model with randomization stratification factors as covariates. The Wilcoxon test for unpaired observations was used to compare groups. Frequencies of individual and combined clinical endpoints on Day 15 and Day 90 are summarized in frequency tables. Logistic regression models were applied using the randomization stratification factors as covariates. In addition, differences between the two treatment groups were tested for statistical significance using Fisher's exact test. Time to cardiovascular event was descriptively analyzed using the Kaplan-Meier method. Median time to cardiovascular event per treatment group and the hazard ratio between the two treatment groups was calculated along with 95% confidence intervals. A Cox regression model was applied for the time to cardiovascular event with randomization stratification factors as covariates. A log-rank test was conducted to test significance between treatment groups.

Table S1. Treatment-Emergent Adverse Events (Safety Population).

	**Active Group (N=25) n (%) Obs**	**Placebo Group (N=22) n (%) Obs**	**Safety Population (N=47) n (%) Obs**
**Any TEAE**	17 (68.0) 39	17 (77.3) 54	34 (72.3) 93
**Cardiac disorders**	6 (24.0) 7	5 (22.7) 8	11 (23.4) 15
Acute myocardial infarction	2 (8.0) 2	0 (0.0) 0	2 (4.3) 2
Atrial fibrillation	1 (4.0) 1	1 (4.5) 1	2 (4.3) 2
**Investigations**	6 (24.0) 7	5 (22.7) 7	11 (23.4) 14
C-reactive protein increased	2 (8.0) 2	3 (13.6) 3	5 (10.6) 5
Hepatic enzyme increased	3 (12.0) 4	2 (9.1) 2	5 (10.6) 6
**General disorders and administration site conditions**	3 (12.0) 4	7 (31.8) 10	10 (21.3) 14
Pyrexia	2 (8.0) 2	2 (9.1) 3	4 (8.5) 5
Edema peripheral	0 (0.0) 0	2 (9.1) 2	2 (4.3) 2
**Infections and infestations**	4 (16.0) 4	4 (18.2) 4	8 (17.0) 8
Pneumonia	2 (8.0) 2	3 (13.6) 3	5 (10.6) 5
**Psychiatric disorders**	3 (12.0) 3	4 (18.2) 4	7 (14.9) 7
Insomnia	1 (4.0) 1	2 (9.1) 2	3 (6.4) 3
**Gastrointestinal disorders**	1 (4.0) 1	4 (18.2) 4	5 (10.6) 5
Diarrhea	1 (4.0) 1	2 (9.1) 2	3 (6.4) 3
**Metabolism and nutrition disorders**	2 (8.0) 3	3 (13.6) 6	5 (10.6) 9
**Nervous system disorders**	3 (12.0) 3	2 (9.1) 2	5 (10.6) 5
Headache	2 (8.0) 2	1 (4.5) 1	3 (6.4) 3
**Skin and subcutaneous tissue disorders**	2 (8.0) 3	1 (4.5) 1	3 (6.4) 4
**Blood and lymphatic system disorders**	0 (0.0) 0	2 (9.1) 3	2 (4.3) 3
Thrombocytopenia	0 (0.0) 0	2 (9.1) 2	2 (4.3) 2
**Injury, poisoning and procedural complications**	1 (4.0) 1	1 (4.5) 1	2 (4.3) 2
**Renal and urinary disorders**	0 (0.0) 0	2 (9.1) 2	2 (4.3) 2
**Respiratory, thoracic, and mediastinal disorders**	1 (4.0) 1	1 (4.5) 1	2 (4.3) 2
**Vascular disorders**	1 (4.0) 1	1 (4.5) 1	2 (4.3) 2
Hypotension	1 (4.0) 1	1 (4.5) 1	2 (4.3) 2

N=number of subjects with events, percentages based on N, Obs=number of events, TEAE=treatment-emergent adverse event. Subjects were counted only once per System Organ Class and Preferred Term
